# Indocyanine green (ICG) fluorescence technology in pediatric robotic surgery

**DOI:** 10.1007/s11701-024-01968-w

**Published:** 2024-05-10

**Authors:** Ciro Esposito, Lorenzo Masieri, Mariapina Cerulo, Marco Castagnetti, Fulvia Del Conte, Claudia Di Mento, Giorgia Esposito, Francesco Tedesco, Roberto Carulli, Leonardo Continisio, Annalisa Chiodi, Maria Escolino

**Affiliations:** 1 Division of Pediatric Surgery, Federico II University Hospital, Via Pansini 5, 80131 Naples, Italy; 2 Division of Pediatric Urology, Meyer Children Hospital, Florence, Italy; 3 Division of Pediatric Urology, Bambino Gesù Children Hospital, Rome, Italy; 4 Division of Internal Medicine, Federico II University Hospital, Naples, Italy; 5 CEINGE Advanced Biotechnology, Naples, Italy

**Keywords:** Indocyanine green, ICG, Robotics, Fluorescence, Technology, Children

## Abstract

This study aimed to report our experience in indocyanine green (ICG) fluorescence-guided surgery (FGS) in pediatric robotics. The data of 55 patients (35 boys and 20 girls), who underwent robotic surgery using ICG fluorescence in three institutions over the last 7 years, were retrospectively reviewed. The following robotic procedures were included: pyeloplasty (*n* = 21), complex Lich–Gregoir ureteral reimplantation (*n* = 8), varicocelectomy (*n* = 7), adnexal pathology resection (*n* = 8), partial nephrectomy (*n* = 4), nephrectomy (*n* = 4), renal cyst removal (*n* = 2), and excision of prostatic utricle (*n* = 1). The ICG was injected intravenously in all indications except for varicocele where intratesticular injection was done, and prostatic utricle or paraureteral diverticulum where trans-catheter injection was done. The ICG dosage was 0.2–0.3 mg/mL/kg. All the procedures were performed using da Vinci Xi platform. Firefly^®^ allowed to switch form bright light to ICG-NIRF view and vice versa. All the procedures were accomplished in robotics without conversions to laparoscopy or open surgery. No episodes of allergy or anaphylaxis to ICG were recorded. An excellent ICG-NIRF view of target organs was obtained in all procedures. Based on our experience, we believe that application of ICG FGS in pediatric robotics enhances the identification of critical anatomical elements and pathological structures, thereby positively impacting both oncological and functional outcomes. This technique is safe, feasible, and versatile. We advocate the consideration of ICG as the standard of care in certain procedures such as partial nephrectomy, varicocele repair, tumor resection, and ovarian torsion. Nonetheless, further investigations are warranted to explore its potential broader applications in pediatric urology.

## Introduction

Over the past decade, there has been a growing utilization of indocyanine green (ICG) fluorescence-guided surgery (FGS) in both adult and pediatric minimally invasive surgery (MIS) procedures [1–6]. The goal is to enhance the intraoperative visualization of anatomical structures, leading to safer and more efficient surgery. ICG fluorescence technology has received approval from the Food and Drug Administration (FDA) and the European Medicines Agency (EMA) for clinical use [7]. More recently, the utilization of ICG fluorescence technology has seen a remarkable surge. This is attributed to the availability of various camera systems from different brands, leading to an exponential increase in its applications in both adults and children [8–10].

From a technical perspective, fluorescence is generated by a specific fluorophore called indocyanine green (ICG) when it is excited using near-infrared (NIR) light, and it is visualized using specific cameras and optics [8–10]. Upon intravenous injection, ICG rapidly binds to various carriers, primarily albumin. Hepatic parenchymal cells almost exclusively take up ICG from the plasma, and the liver completely secretes it into the bile. For visualizing lymphatic vessels, ICG is administered into the target organs or the peritumoral area. In cases involving the urinary tract, ICG can be introduced into a Foley catheter to assess bladder morphology and any malformations, or it can be inserted into ureteral catheters to identify the ureters [11, 12]. To use ICG near-infrared fluorescence (NIRF) in MIS, an ICG vial, specialized optics, and cameras are required. In robotic surgery, using the da Vinci Xi platform, ICG technology is already integrated into the system (Firefly^®^, Novadaq^®^ Technologies Inc., Toronto, Canada) [13].

This study aimed to share our insights gained from employing ICG fluorescence technology in robotic procedures to perform intraoperative anatomic navigation and enhance our decision-making processes during complex surgical procedures.

## Materials and methods

The data of 55 patients, who underwent robotic surgery using ICG-NIRF assistance in three different pediatric surgery units over the last 7 years, were retrospectively reviewed. Patients included 35 boys and 20 girls, with a median age of 8.5 years (range 1–17). The patients underwent the following robotic procedures with ICG-NIRF assistance: pyeloplasty (*n* = 21), complex Lich–Gregoir ureteral reimplantation (*n* = 8), varicocelectomy (*n* = 7), resection of adnexal pathology (*n* = 8), partial nephrectomy (*n* = 4), nephrectomy (*n* = 4), renal cyst removal (*n* = 2), and excision of prostatic utricle cyst (*n* = 1).

ICG was injected intravenously for all indications except for varicocele and prostatic utricle. The ICG dosage was 0.2–0.3 mg/mL/kg. All the procedures were performed using the da Vinci Xi platform with a double console system. The Firefly^®^ system allowed to easily switch form bright light view to ICG-NIRF view and vice versa. Dosage and modality of administration of ICG in the different indications are described below.

### Operative procedures

#### Varicocelectomy

**Fig. 1:**
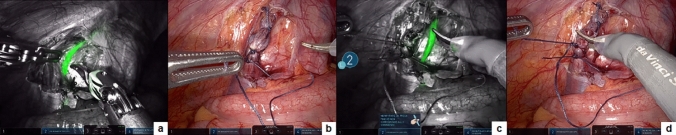
Robotic varicocelectomy: lymphatic sparing on ICG-NIRF (**a**); spermatic vessel ligation (**b**) with lymphatic preservation (**c**); spermatic vessel division (**d**)

Following the creation of a pneumoperitoneum and the placement of ports comprising three 8 mm robotic ports and one 5 mm assistant port, the robot was docked to the patient in accordance with the lower abdominal specific setup. After completing the dissection of the spermatic bundle, a 1 mL solution of 2.5 mg/mL ICG was injected into the body of the left testis using a 23-gauge needle, and Firefly^®^ was activated. Within 60 s of the intratesticular injection, ICG-NIRF became evident in the lymphatic vessels, which were readily visible and spared. Subsequently, the remaining steps of the procedure, including the clipping and sectioning of the spermatic bundle according to the Palomo procedure, were successfully executed (Fig. 1).

#### Pyeloplasty/nephrectomy/renal cyst removal

**Fig. 2:**
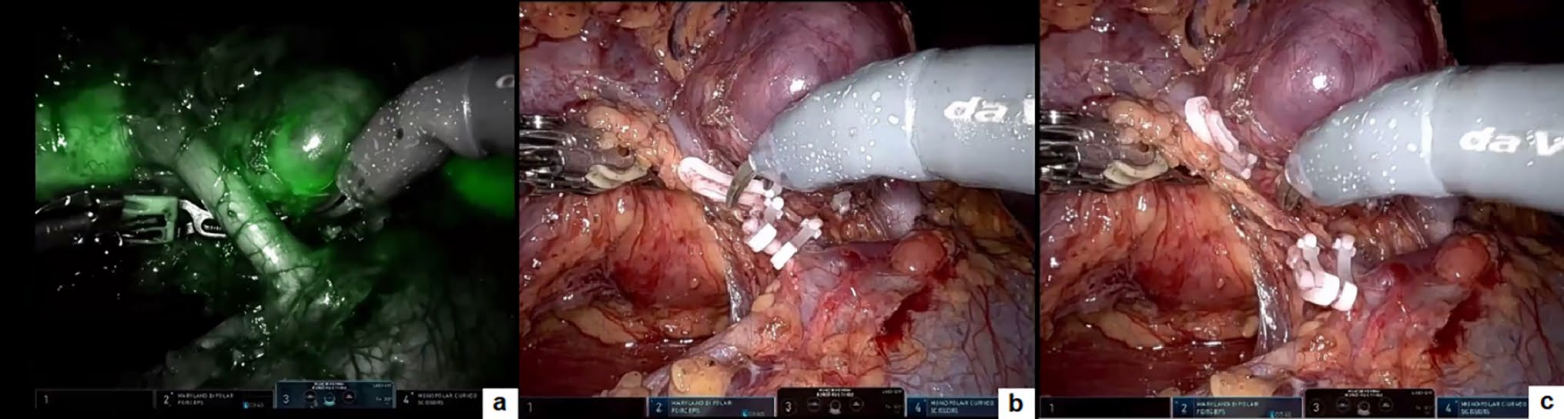
Robotic nephrectomy: visualization of renal hilum aided by ICG-NIRF (**a**); clipping (**b**) and division (**c**) of the renal vein

Following the creation of a pneumoperitoneum and the placement of ports comprising three 8 mm robotic ports and one 5 mm assistant port, the robot was docked to the patient in accordance with the right/left lateral (renal) specific setup. After opening of the Gerota fascia and prior to the renal hilum dissection, a bolus of 0.3 mg/mL/kg ICG solution was administered via a peripheral vein. Within 30–120 s, ICG-NIRF became visible in the renal vascular pedicle. This proved to be very helpful in identifying vascular anomalies or crossing vessels, particularly in cases with distorted anatomy due to prior inflammation (Fig. 2). In case of simple renal cysts, ICG-NIRF aided in identifying the cyst dome, which exhibited reduced fluorescence compared to the surrounding healthy renal parenchyma. This facilitated the performance of an anatomical and nearly complete resection of the cyst roof.

#### Partial nephrectomy

Following the creation of a pneumoperitoneum and the placement of ports comprising three 8 mm robotic ports and one 5 mm assistant port, the robot was docked to the patient in accordance with the right/left lateral (renal) specific setup. ICG was administered in three distinct steps. The first injection of 10 mL of 2.5 mg/mL ICG solution was carried out via the ureteral catheter, which had been placed through cystoscopy into the ureter of the normally functioning moiety. This enabled the identification of the normal ureter, which exhibited green fluorescence on ICG-NIRF, facilitating differentiation from the pathologic and dilated ureter of the non-functioning moiety. Subsequently, a second injection of a bolus of 0.3 mg/mL/kg ICG solution was administered intravenously to conduct the angiographic phase. This allowed visualization of the main hilar vessels and the small vessels supplying the non-functioning moiety. Finally, a third injection of a bolus of 0.3 mg/mL/kg ICG solution was repeated intravenously following the clipping and division of the vessels supplying the kidney moiety. This served a dual purpose: to visualize the demarcation line between the ischemic moiety to be removed (not colored) and the perfused healthy renal moiety, and to assess the perfusion of the remaining moiety after parenchymal resection of the non-functioning pole.

#### Complex Lich–Gregoir ureteral reimplantation

**Fig. 3:**
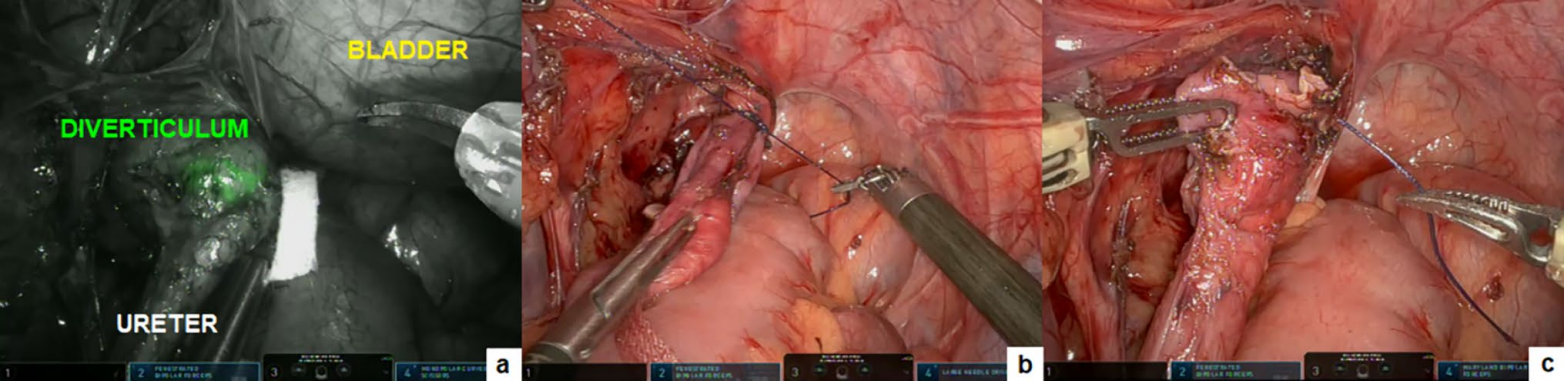
Robotic treatment of paraureteral diverticulum: ICG-NIRF facilitated the precise delineation of the point of entrance of the diverticulum into the bladder (**a**), aiding in its ligation (**b**) and division (**c**)

Following the creation of a pneumoperitoneum and the placement of ports comprising three 8 mm robotic ports and one 5 mm assistant port, the robot was docked to the patient in accordance with the pelvic-specific setup. ICG-NIRF was preferentially employed in cases involving complex vesicoureteral reflux (VUR) associated with paraureteral diverticulum. For such indication, two injections of ICG were given. Firstly, a 5–10 mL injection of 2.5 mg/mL ICG solution was administered via the ureteral catheter, which had been inserted through cystoscopy into the diverticulum. This facilitated the precise delineation of the point of entrance of the diverticulum into the bladder, aiding in its clear dissection and ligation (Fig. 3). Subsequently, a second injection of a bolus of 0.3 mg/mL/kg ICG solution was delivered intravenously after detaching the dilated ureter from the bladder and following ureteral tapering to evaluate ureter perfusion.

#### Resection of adnexal pathology

In gynecological indications, three robotic arms were utilized: one 12 mm arm equipped with a 12–8 mm reducer to accommodate the 0-degree robotic optic, and two 8 mm ports to house the robotic instruments. Additionally, a fourth 5 mm accessory port was inserted. The robot was then docked over the patient’s feet, aligning with the pelvic (gynecologic)-specific setup. Robotic vessel sealer technology was employed throughout.

**Fig. 4:**
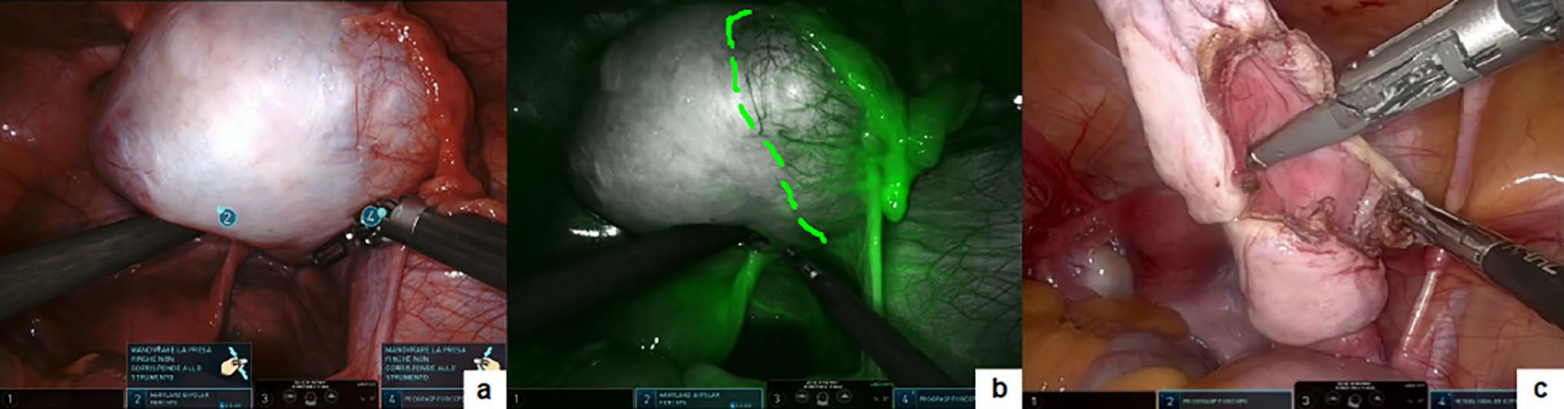
Robotic treatment of ovarian mass: ICG-NIRF allowed to assess the resection margin (**a, b**) and decide on ovarian-sparing surgery (**c**)

**Fig. 5:**
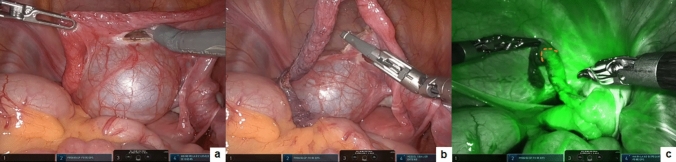
Robotic excision of giant paratubal lesion: following the removal of the paratubal lesion (**a, b**), ICG-NIRF aided to assess the perfusion pattern of the fallopian tube (**c**)

During surgery, ICG was administered intravenously at a dosage of 0.5 mg/mL/kg. Within a span of 90 s, fluorescence emerged in the target organs, facilitating the identification of the resection margins and the assessment of perfusion. ICG-NIRF was applied in ovarian masses to assess resection margins and guide intraoperative decision-making (Fig. 4), as well as in adnexal torsion or paratubal lesion to assess the perfusion patterns before and after detorsion or excision and decide for conservative treatment (Fig. 5).

#### Excision of prostatic utricle cyst

Under general anesthesia, initial cystourethroscopy confirmed the diagnosis of a utricular remnant and identified its orifice on the verumontanum. A ureteral catheter was then inserted into the utricular cyst and left in place. Following the creation of a pneumoperitoneum and the placement of ports, including three 8 mm robotic ports and one 5 mm assistant port, the robot was positioned in accordance with the pelvic-specific setup. At this step, 2.5–3 mL of 2.5 c/mL ICG solution were injected directly into the utricular cyst via the ureteral catheter. The resulting ICG-guided fluorescence facilitated clear visualization of the cyst and aided in its meticulous dissection between the bladder and rectum using a combination of monopolar and bipolar energy for safe ligation of its communication to the urethra. Subsequently, after freeing it from surrounding tissues, the cyst neck was ligated with a loop and excised. Finally, the cyst was retrieved through the umbilical port.

## Results

All procedures were successfully completed using robotics without any conversions to laparoscopy or open surgery. The median docking time was 12 min, with a range of 8–22 min.

Regarding the timing of intraoperative visualization of ICG-NIRF, in patients undergoing Palomo varicocelectomy, the median time for lymphatic visualization was 60 s, ranging from 30 to 120 s following the intratesticular injection. The lymphatics remained clearly visible for at least 5–7 min. Subsequently, a diffusion of the dye was observed in the entire spermatic bundle, and the lymphatics were no longer distinctly visible. However, the median time to spare the lymphatics was 1–2 min, allowing lymphatic sparing in all operated patients. In cases of intravenous administration, such as in renal procedures, the median time for visualization of ICG-NIRF in the target organs was 60 s, with a range of 30–120 s following the intravenous injection. In case of trans-catheter injection, ICG-NIRF was immediately visible in the target structures such as the ureter, the diverticulum, or utricle remnant.

No episodes of allergy or anaphylaxis to the ICG were recorded. An excellent ICG-NIRF view of target organs was achieved in all procedures. No intraoperative complications occurred. The median length of stay was 2.4 days, ranging from 1 to 5 days. No postoperative complications were recorded in any patient.

Patient demographics and outcome parameters are reported in Table 1.

**Table 1:** Patient demographics and outcome parameters

Parameter	Varicocelectomy	Pyeloplasty	Renal cyst removal	Nephrectomy	Partial nephrectomy	Complex ureteral reimplantation	Resection of adnexal pathology	Excision of prostatic utricle cyst
Patients, *n*	7	21	2	4	4	8	8	1
M/F*, n/n*	7/0	17/4	2/0	3/1	2/2	3/5	0/8	1/0
Median age, years (range)	14.5 (12–17)	4.7 (1–9)	8.5 (7–15)	13.5 (8–16)	5.4 (3–7)	6.7 (5–11)	14.7 (11–16)	4
Median weight, kg (range)	66.5 (40–80)	22.5 (13.8–35)	38 (28–67)	51 (32–68)	28.7 (19–32)	33.6 (25–48)	56.6 (38–69)	20
Operative time*, *min (range)	34.5 (30–46)	123 (95–180)	75 (64–89)	85 (70–95)	110 (87–155)	106 (96–170)	42 (23–68)	115
Docking time, min (range)	10 (8–15)	16 (13–22)	13.5 (14–21)	11.5 (12–20)	12.5 (9–18)	10.3 (8–15)	11.2 (9–17)	11
Intraoperative complications, n (%)	0	0	0	0	0	0	0	0
Conversion, n (%)	0	0	0	0	0	0	0	0
ICG dosage	1 mL of 2.5 mg/mL ICG solution	0.3 mg/mL/kg	0.3 mg/mL/kg	0.3 mg/mL/kg	1) 10 mL of 2.5 mg/mL ICG solution 2) 0.3 mg/mL/kg	1) 5–10 mL of 2.5 mg/mL ICG sol 2) 0.3 mg/mL/kg	0.5 mg/mL/kg	2.5–3 mL of 2.5 mg/mL ICG solution
ICG administration route	Intratesticular	Intravenous	Intravenous	Intravenous	1) Trans-catheter 2) Intravenous	1) Trans-catheter 2) Intravenous	Intravenous	Trans-catheter
Timing of ICG-NIRF visualization, s (range)	60 (30–120)	60 (30–120)	60 (30–120)	60 (30–120)	60 (30–120)	0	60 (40–80)	0
Postoperative complications, *n* (%)	0	0	0	0	0	0	0	0
Median LOS, days (range)	1.1 (0–1.5)	3.8 (3–5)	2.3 (2–4)	2.2 (2–4)	3.3 (2–5)	2.7 (2–4)	1.3 (1–2)	2.5
Allergy or systemic adverse reaction to ICG, *n *(%)	0	0	0	0	0	0	0	0

## Discussion

In the last decade, two significant innovations have emerged in pediatric surgery and urology: the adoption of robotic surgery to address pediatric surgical conditions and the utilization of FGS with ICG [5, 6, 14, 15]. Our teams were among the pioneers in both techniques, having initiated the use of robotics approximately 19 years ago [16] and incorporating ICG technology into our practice over the last 7 years [17, 18]. Due to our extensive experience in these areas, we aimed to report in the present study our 7 year journey with ICG fluorescence technology in robotic surgery using the da Vinci Xi platform.

We believe that the main objective of any surgical procedure is to attain optimal surgical outcomes. To achieve this goal, the visualization and identification of significant and critical anatomical structures are paramount. According to our experience, the utilization of ICG fluorescence represents an effective approach to achieve this goal, as it facilitates enhanced visualization of these structures during surgery [5, 17]. ICG technology has seen widespread adoption by adult surgeons over the past decade, particularly in biliary surgery [19], and more recently, in pediatric laparoscopy [5, 6]. However, the published literature on the application of ICG fluorescence imaging in robotic surgery, especially within the pediatric field, is still weak [18, 20, 21].

ICG was developed for NIR photography by Kodak Research Laboratories in 1955 and has been in clinical use since 1956 [17]. Initially, ICG was employed to measure cardiac output, study the anatomy of retinal vessels, and assess residual liver function in liver cirrhosis before liver resection. It can be injected into the human bloodstream with no adverse effects. Upon excitation with light at a specific wavelength in the NIR spectrum emitted from a particular light source or NIR laser device, ICG becomes fluorescent. This fluorescence can be captured by dedicated optics and cameras and then displayed on a video monitor, enabling visualization of the anatomical structures of interest where the dye has accumulated, such as bile ducts, vessels, lymph nodes, lymphatics, tumors, and organs like kidney or spleen [8–10].

In our robotic surgical practice, we have successfully implemented ICG fluorescence technology for various pathologies and indications with consistently excellent outcomes. One notable application is in the treatment of varicocele, where the lymphatic-sparing Palomo procedure is commonly performed in pediatric surgery. Robotic-assisted varicocelectomy using ICG fluorescence guidance proved to be an excellent approach for this procedure [22, 23]. The robotic instruments' dexterity allows for precise identification and sparing of lymphatics after visualization with ICG-NIRF. Additionally, robotics allows to avoid the use of clips, as it facilitates the easy ligation of the spermatic vessels.

Partial nephrectomy represents another excellent indication for utilizing ICG fluorescence technology [21, 24]. This typically involves three injections: the first injection is administered through a ureteral catheter positioned via cystoscopy in the normal ureter, facilitating the identification and differentiation of the normal ureter from any pathologic ureter. The second step involves an intravenous injection to identify the vascularization of the two kidney moieties. The third injection, also administered intravenously, is utilized to delineate the resection line between the normal moiety and the non-functioning moiety needing removal.

Furthermore, ICG fluorescence technology can be beneficial in other renal procedures [11]. For nephrectomy, it aids in identifying renal vessels, particularly in cases of strong adhesions post-pyelonephritis or giant renal cysts, where the cyst dome remains uncolored after injection [25]. In the context of pyeloplasty for ureteropelvic junction obstruction, ICG fluorescence can be helpful in cases where there is suspicion of extrinsic obstruction due to crossing vessels. It facilitates the identification of such vessels and ensures the correct vascularization of the ureter after isolation and spatulation, before performing the anastomosis. For bladder, ureteral, and urethral pathologies, ICG fluorescence technology offers valuable guidance during robotic surgeries. In cases of prostatic utricle, ICG solution can be injected through a ureteral catheter into the utricle, allowing green fluorescence to guide the surgeon during robotic dissection. Similarly, for bladder diverticula associated with VUR, ICG can be injected into the diverticulum via the same technique used for the utricle, aiding in dissection. In ureteral reimplantation procedures, ICG can also be utilized to assess ureteral vascularization, particularly in cases of megaureter needing tapering, before reimplantation into the bladder. Furthermore, in cases of renal or ovarian tumors, ICG can be employed to identify tumor vascularization and assess node involvement, providing valuable information during surgical planning and execution [26–28].

ICG-enhanced fluorescence imaging holds significant utility in pediatric and adolescent gynecologic conditions, extending beyond cancer to encompass various benign pathological conditions and emergencies [29]. Specifically, in cases of ovarian torsion, intravenous injection of ICG enables visualization of the ovarian vascular network and the exact point of torsion in the ovarian vessels. Following ovarian detorsion, ICG fluorescence imaging proves very valuable in assessing the vascularization of the ovary, aiding in post-torsion evaluation and management [26, 30].

One criticism we have regarding this technique is related to the ICG software on the da Vinci Xi platform. When transitioning from normal view to ICG view, the screen turns black and white. Future robotic platforms should adopt a new system, where the screen retains color during this transition. This enhancement would provide a more seamless and user-friendly experience during ICG-enhanced procedures.

## Conclusion

Based on our experience in pediatric robotics, we strongly affirm that ICG technology significantly enhances the identification of critical anatomical elements and pathological structures, thereby positively impacting both oncological and functional postoperative outcomes. This technique is not only safe and non-toxic, but also feasible to perform and versatile. We advocate the consideration of ICG as the standard of care in certain procedures such as partial nephrectomy, varicocele repair, tumor resection, and ovarian torsion. Nonetheless, further investigations are warranted to explore its potential broader applications in pediatric urologic operations.

## Data Availability

All data will be available on request.
